# Non-Human Primates Harbor Diverse Mammalian and Avian Astroviruses Including Those Associated with Human Infections

**DOI:** 10.1371/journal.ppat.1005225

**Published:** 2015-11-16

**Authors:** Erik A Karlsson, Christopher T Small, Pamela Freiden, MM Feeroz, Frederick A Matsen, Sorn San, M Kamrul Hasan, David Wang, Lisa Jones-Engel, Stacey Schultz-Cherry

**Affiliations:** 1 Department of Infectious Disease, St Jude Children’s Research Hospital, Memphis, Tennessee, United States of America; 2 Computational Biology Program, Fred Hutchinson Cancer Research Center, Seattle, Washington, United States of America; 3 Department of Zoology, Jahangirnagar University, Savar, Bangladesh; 4 National Veterinary Research Institute, Phnom Penh, Cambodia; 5 Department of Pathology & Immunology, Washington University, St. Louis, Missouri, United States of America; 6 University of Washington, National Primate Research Center, Seattle, Washington, United States of America; Cincinnati Children’s Hospital Medical Center, UNITED STATES

## Abstract

Astroviruses (AstVs) are positive sense, single-stranded RNA viruses transmitted to a wide range of hosts via the fecal-oral route. The number of AstV-infected animal hosts has rapidly expanded in recent years with many more likely to be discovered because of the advances in viral surveillance and next generation sequencing. Yet no study to date has identified human AstV genotypes in animals, although diverse AstV genotypes similar to animal-origin viruses have been found in children with diarrhea and in one instance of encephalitis. Here we provide important new evidence that non-human primates (NHP) can harbor a wide variety of mammalian and avian AstV genotypes, including those only associated with human infection. Serological analyses confirmed that >25% of the NHP tested had antibodies to human AstVs. Further, we identified a recombinant AstV with parental relationships to known human AstVs. Phylogenetic analysis suggests AstVs in NHP are on average evolutionarily much closer to AstVs from other animals than are AstVs from bats, a frequently proposed reservoir. Our studies not only demonstrate that human astroviruses can be detected in NHP but also suggest that NHP are unique in their ability to support diverse AstV genotypes, further challenging the paradigm that astrovirus infection is species-specific.

## Author Summary

With the advances in next generation sequencing and pathogen discovery, astrovirus (AstV), leading cause of diarrhea in children, the elderly and immunocompromised people, detection in diverse animal hosts has increased. Yet, to date there has been no detection of AstVs associated with human infections in animals suggesting these strains are specific to humans. In these studies we demonstrate that non-human primates (NHP) harbor a wide variety of AstVs including those previously only detected in people. Further, we identified an NHP with an AstV that is a recombination between human and animal genotypes. Our studies provide important new evidence that human astroviruses can be detected in animals directly challenging the paradigm that AstV infection is species-specific.

## Introduction

Astroviruses (AstV) are small, non-enveloped, positive-sense, single-stranded RNA viruses associated with significant morbidity, especially in the young, elderly and immunocompromised people as well as substantial economic losses in poultry [1,2]. Although most commonly associated with diarrhea, they can also cause a variety of clinical diseases including nephritis, hepatitis, and encephalitis or can be asymptomatic depending on the species. Since 2008, the number of animal hosts shown to be infected with AstVs has quadrupled to include at least 30 mammalian and 14 avian species [3,4] with a correlative increase in genetic diversity resulting in division of the *Astroviridae* family into two genera, *Mamastrovirus* (MAstVs) and *Avastrovirus* (AAstVs) that are further sub-divided into genotypes or viral species based on the genetic differences within the complete viral capsid protein [5]. However, with the constant identification of new viral species and hosts, and the genetic diversity within the family, it is likely that the *Astroviridae* family will continue to diverge and taxonomy and nomenclature will have to be updated regularly.

AstV infections are thought to be species-specific [2,3,5,6]. Yet, phylogenetic characterization suggests that a single host species may be susceptible to infection with divergent AstV genotypes. For example, humans can be infected with the “classical” serotypes HAstV1-8 or the recently identified HAstV-MLB1-3, HMO AstVs A, B, and C, and HAstV-VA1-4 viruses [6,7]. These recently identified human AstVs are genetically much closer to AstVs from animals than they are to the canonical HAstVs. Similar observations were reported for AstVs detected in pigs, bats, California sea lions, sheep, mink, and turkeys [8] challenging the paradigm that AstV infections are species-specific. Indeed, recent studies have shown a mammalian-like virus in an avian host [9]. Yet to date, diverse MAstV and AAstV genotypes, especially viruses associated with human infections have not been detected in a single animal host. However, potential human-mammalian recombination events have been detected suggesting that the species barrier may have been crossed at some point [10,11].

Non-human primates (NHP) are highly susceptible to a variety of enteric viruses [12–15]. In Bangladesh, rhesus macaques, which are ubiquitous and often synanthropic (i.e. species that thrive in human-altered habitats) were shown to be infected with a variety of human enterovirus serotypes that shared considerable genetic overlap with viruses detected in closely associated humans, strongly suggesting interspecies transmission [13]. There is also serological evidence suggesting natural infection with rotavirus and norovirus among captive NHP [16]. No data are currently available on AstVs in NHP [13,15]. The objective of this study was to fill this gap in knowledge and determine the extent of AstV among NHP populations in Bangladesh and Cambodia.

In Bangladesh and Cambodia, multiple species of NHP including rhesus macaques *(Macaca mulatta*), Hanuman langurs (*Semnopithecus entellus*), longtailed macaques (*M*.*fascicularis*) and pigtailed macaques (*M*.*nemestrina*) have for centuries thrived at the human-primate interface, ranging freely through villages and religious sites [17,18]. These macaques and langurs, as well as species of gibbons (*Hylobates spp*.), are also found in captive settings. We have found evidence, based on analysis of sequences derived from the highly conserved RNA-dependent RNA polymerase (RdRp) gene, that these NHP harbor a variety of MAstV, including genotypes previously only associated with human infections, sequences with little similarity to currently identified AstVs,. AAstV genotypes, and what appears to be a recombinant between a human AstV and unique virus hitherto only detected in NHPs. We contrast this diversity with that observed in bats, which have been identified as having exceptionally diverse AstV populations (14–16), but which our studies indicate is more phylogenetically isolated from AstV infecting other mammals. Importantly, the presence of antibodies to HAstVs further supports our hypothesis that NHP are susceptible to infection with human astrovirus genotypes. These studies provide important new evidence that primates can be infected with human astroviruses. They also directly challenge the paradigm that AstV infection is species-specific.

## Results

### Human AstV genotype detection in NHP

**Fig 1:**
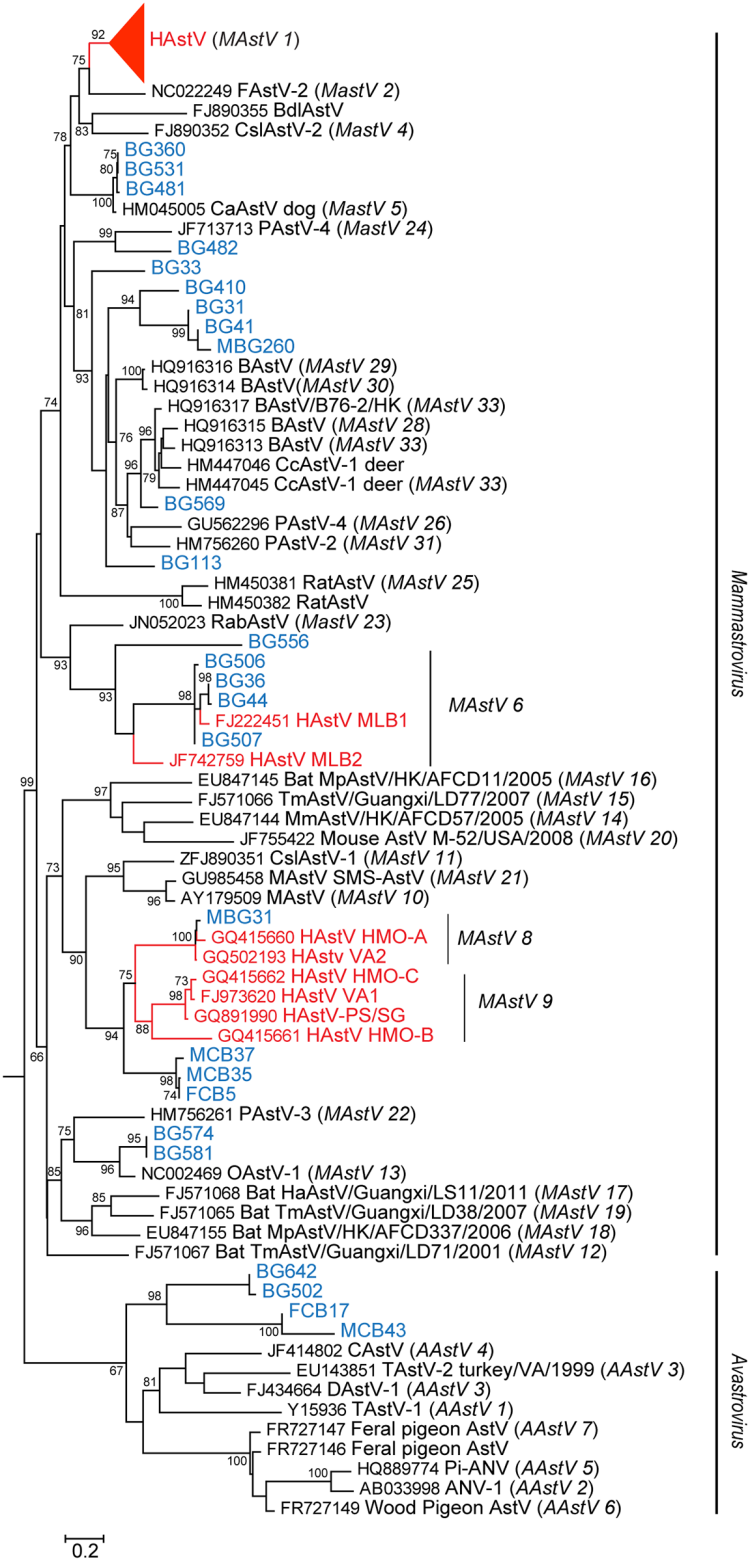
NHPs harbor diverse AstV genotypes. RdRp sequences were aligned using MAFFT v7.058b and phylogenetic trees were constructed and evolutionary history inferred using the Neighbor-Joining method in MEGA6. GenBank accession numbers for the reference strains are given before the strain name and assigned or putative (in italics) AstV genogroups listed in parenthesis. Human viruses are in red and NHP samples in blue.

**Fig 2:**
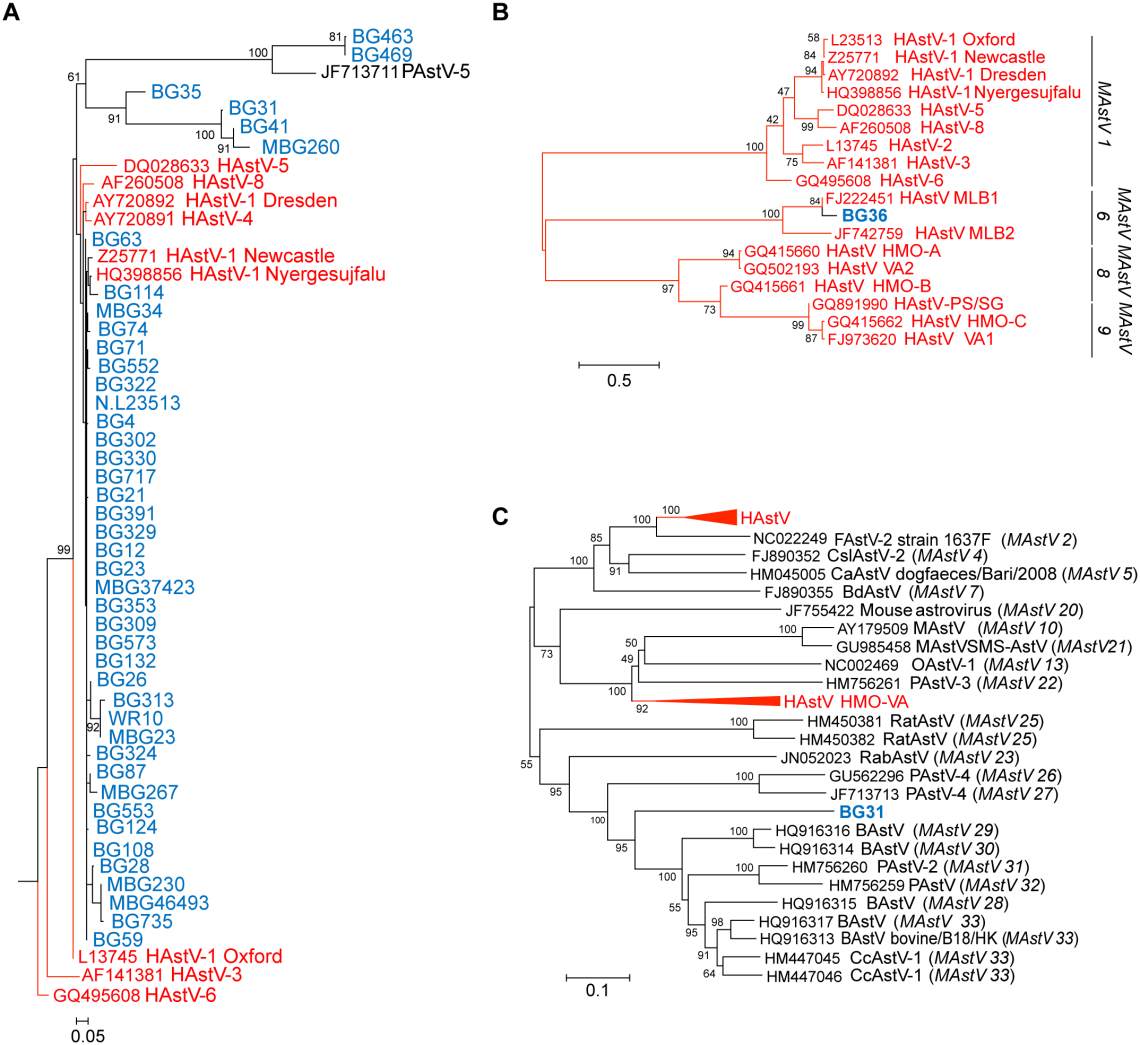
NHPs harbor AstV genotypes associated with human infections. (A) Magnified HAstV clade from Fig 1. (B-C) Clustal W alignments on ~300 nucleotides from the ORF2 capsid gene of NHP BG36 (B) or ~900 nucleotides of NHP BG31 (C) were performed using BioEdit and MEGA6. Phylogenetic trees were constructed and evolutionary history inferred using the Neighbor-Joining method. GenBank accession numbers for the reference strains are given before the strain name and assigned AstV genogroups listed. Human viruses are in red and NHP in blue.

Fecal samples from NHP in Bangladesh and Cambodia were collected between 2007–2008 and 2011–2012 and RNA screened using a pan-astrovirus RT-PCR targeting a 422 nucleotide segment within the highly conserved RNA-dependent RNA polymerase (RdRp) gene [19]. Of the 879 fecal samples tested 68 (7.7%) were AstV positive (Table 1). S1 Table contains the complete details on the positive samples including NHP species, percent similarity to closest identified sequence, and proposed nomenclatures. Sequence analysis unexpectedly revealed that HAstV, MLB, and VA genotypes were detected in NHPs (Fig 1). The majority of the positive samples (60.3%) were 98–100% similar to HAstV-1 reference viruses (Fig 2A) and 11.7% of the samples were 79 to 84% similar to human VA and MLB reference viruses (Fig 1). Intriguingly, MLB and VA sequences were detected in NHP samples collected in 2007 prior to the official identification in 2008 and 2009 respectively [10,20,21]. Although most of the human-like sequences were closely related to human reference sequences, NHP FCB5, MCB35, MCB37, which were collected in Cambodia in 2011–2012, branched off the human VA/HMO subclade forming a unique clade.

**Table 1:** Diverse AstV genotypes detected in NHP samples.

Country	Bangladesh			Cambodia		
AstV Genotype	Human	Mammalian	Avian	Human	Mammalian	Avian
Context						
Pet/Performing	3/23 (13%)	ND^1^	ND	ND	ND	ND
Temple	15/229 (6.6%)	3/229 (1.3%)	ND	ND	ND	2/27 (7.4%)
Urban	21/458 (4.6%)	10/458 (2.2%)	ND	3/16 (18.8%)	ND	ND
Tea Garden/Park	2/16 (12.5%)	1/16 (6.3%)	ND	ND	0/5 (0%)	ND
Zoo/Captive	2/86 (2.3%)	ND	2/86 (2.3%)	1/21 (4.8%)	ND	ND
Wild	3/29 (10.3%)	1/29 (3.4%)	ND	ND	ND	ND

^1^Not Detected

Numerous approaches including genome walking, 3’RACE, and deep sequencing were undertaken on all RdRp-positive samples to obtain more genomic information. We obtained ~300 nucleotides from the 5’ end of MAstV/Hoolock gibbon/Bangladesh/BG36/2007 ORF2 and confirmed that it was 84% similar to MLB1 capsid sequences (Fig 2B). These results suggest that canonical (HAstV1-8) and non-canonical (MLB, VA/HMO) viruses can be detected in NHP.

### Serological support that NHP are infected with AstVs associated with human infection

Given that human AstVs have not been previously detected in mammals, we tested NHP sera for the presence of antibodies against HAstV-1, and MLB capsid proteins by ELISA [22,23]. Turkey astrovirus type-2 (TAstV-2) is genetically distant from the mammalian viruses and we found no evidence of TAstV-2-like sequences in our genetic analysis, thus it was used as an AstV “control” (Fig 1). Briefly, 96-well plates were coated with purified recombinant HAstV-1, MLB1, MLB2, TAstV-2 capsid proteins or BSA and limiting dilutions of the NHP sera was incubated as described [22,23]. Positive controls included known positive polyclonal antisera and human sera. Unfortunately reagents to other AstV genotypes are unavailable. Of the sera samples available for testing, 90 from Bangladesh and 48 from Cambodia, 44 (31.9%) samples were positive for AstV antibodies with the majority (72.7%) specific for HAstV-1 and 27.3% positive for the non-canonical MLB capsids (Table 2). Cross-reactive MLB1 and MLB2 antibodies have been detected by ELISA so these results were combined although sera was run against MLB1 and MLB2 capsids [23]. None of the samples were positive for TAstV-2. Eighty-five of the sera were from NHP that had also been tested for AstV by RT-PCR. Of the 22 serologically positive sera, 7 were from NHP that were also RT-PCR positive (S1 Table). From these seven NHP, we identified three primates that were both serologically and RT-PCR positive: NHP MBG248 was serologically positive for MLB and RT-PCR positive for HAstV-1; MCB35 was serologically and RT-PCR positive for HAstV-1; and MCB43 was serologically HAstV-1 positive while RT-PCR positive for avian AstV. Combined with our genetic data, these studies strongly suggest that NHP not only harbor human AstV strains but have antibodies suggestive of previous exposures.

**Table 2:** Antibodies against human AstV strains were detected in NHP sera.

ELISA	Number Positive	Percent of Total	Percent of Positive
HAstV1	32	23.2%	72.7%
MLB	12	8.7%	27.3%
TAstV-2	0	0.0%	0.0%
Negative	94	68.1%	
Total Tested	138		

### Detection of diverse mammalian and avian AstVs genotypes in NHP

In addition to the human-like sequences, 23.5% of the samples were similar to MAstVs isolated from diverse animal hosts including dogs, pigs, and sheep (Fig 1). NHP BG33, BG113, BG569, and BG410 and its related subclade containing BG31, BG41, and MBG260 were collected in Bangladesh in 2007–2008 and 2012 (MBG260) and appear to be part of a larger cluster of viruses identified in cows, pigs, and deer (Fig 1). We were able to obtain ~900 nucleotides of MAstV/Rhesus macaque/Bangladesh/BG31/2007 ORF2 and confirmed that it clusters within the same clade (Fig 2C). NHP BG463 and BG469 cluster with a unique porcine AstV (PAstV-5) [24] forming a poorly supported subclade off the HAstVs (Fig 2A).

Additionally, 4.4% of the positive samples clustered within the AAstVs though they formed a distinct and well supported subclade from previously identified AAstV genotypes (Fig 1). Although we demonstrated that people with occupational exposure to poultry can have antibodies against AAstVs [22], only one prior study has successfully isolated AAstV from a mammal [25]. Overall, these data demonstrate that NHP can harbor a variety of mammalian including human and avian AstVs. Unfortunately, attempts to isolate the NHP viruses or obtain further genomic data either by traditional or deep sequencing methodologies were unsuccessful.

### Evidence of recombination

Recombination events have been detected in numerous MAstV and AAstVs and are thought to be a major factor in the evolution of *Astroviridae* [4,5,10,11,21]. They can also confound phylogenetic reconstruction. Indeed, when constructing the RdRp phylogenetic tree, inclusion of the NHP BG35 sequence in the alignment resulted in a unique subclade that branched off the HAstVs (Fig 2A). Included within this subclade were NHP BG31, BG41, and MBG260, sequences that were shown to cluster within the cows, pigs, and deer RdRp (compare Fig 1) when NHP BG35 is excluded from the alignment. Given that the BG31 capsid sequence was clearly shown to align within this larger cow, pig, deer clade (Fig 2C), we hypothesized that NHP BG35 was a possible recombinant AstV.

**Fig 3:**
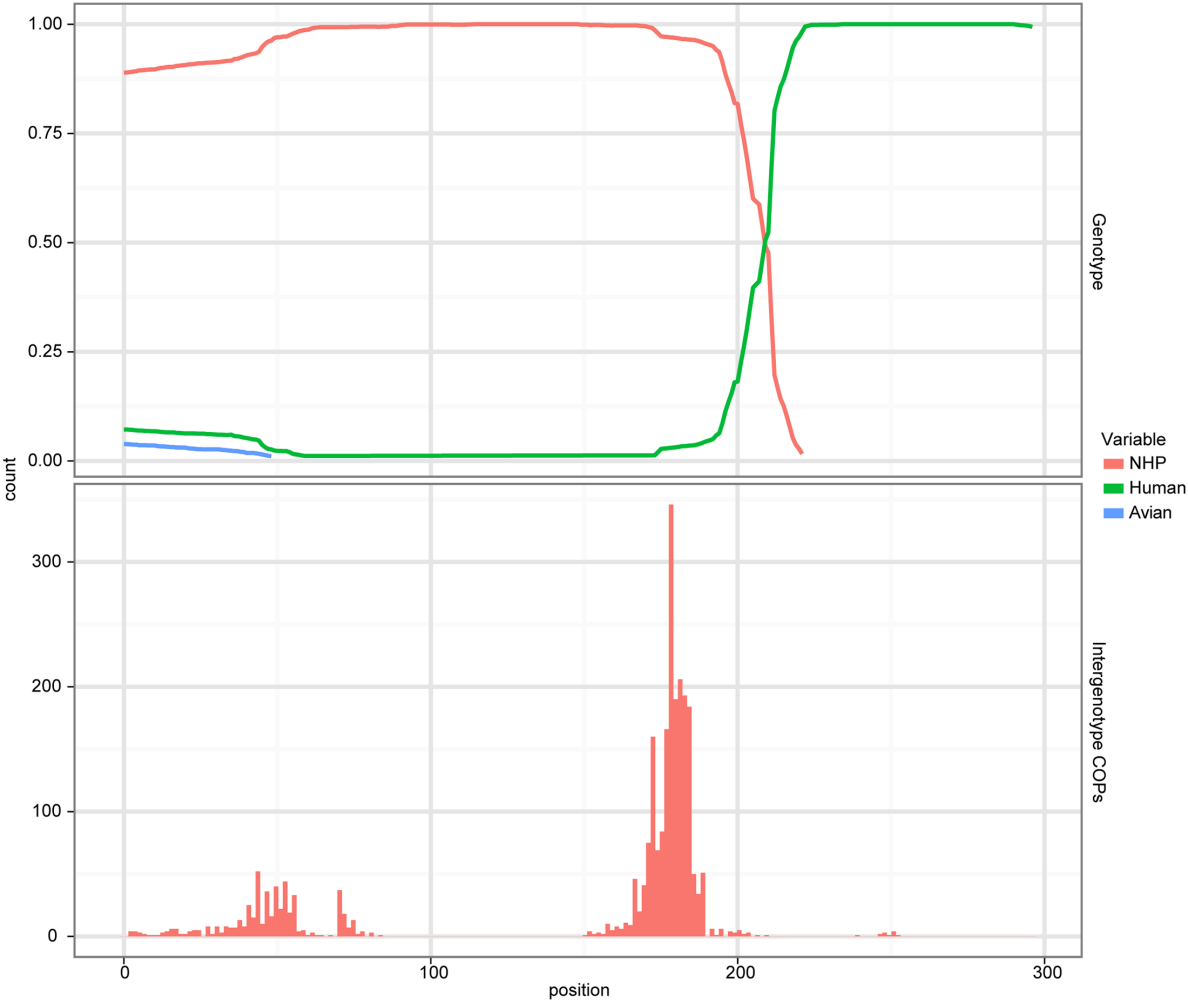
Evidence of recombination. A cBrother analysis established the recombinant relationship between the human AstV lineage represented by sequence N.L23513 and the NHP AstV lineage represented by the sequence from BG31. Both the top and bottom panels share a common X-axis representing the position within the trimmed alignment. The top panel represents the posterior probability of ancestral assignment for the corresponding ancestral line at a given position within the alignment. The bottom panel represents the number of crossover points which occur at a given alignment position out of the 1000 samples taken from the MCMC chain.

To test this hypothesis, the sequences, which upon visual inspection best matched the 5’ and 3’ end of the NHP BG35, were identified as the canonical human AstVs and the NHP viruses phylogenetically close to BG31, respectively. To represent these two putatively parental genotypes in recombination analyses, we chose the HAstV-2 sequence L23513 and the NHP AstV BG31. The sequence from BG31 in particular was chosen because it provided the best overlap with the region of interest in BG35. The global alignment was subset to these two sequences, together with the putative recombinant sequence and the duck AstV sequence FJ434664 as an out group. This alignment was further trimmed using trimal [26] with settings -gt 0.25 -sw 3 to reduce the effect of gap positions on the analysis, resulting in a 297 bp alignment. This resulting alignment tested positive for recombination via the Phi test [27], as implemented in PhiPack (P = 9.3x10^-6^) (Fig 3).

To verify the specific nature of the recombination, a cBrother analysis was performed using the same alignment used in the Phi test. The duck, human and BG31 AstV sequences were used as genotype representatives in this analysis. Two independent cBrother runs of 1.1 million generations were run, with the initial 10% discarded as burn-in, and sampling every 1000 generations. Convergence was assessed using the Gelman–Rubin diagnostic [5] included with the cBrother distribution. This analysis found a recombinant breakpoint at base 209 of the subset alignment, with ancestry assigned to the BG31 genotype on the 5’ side, and to the human AstV on the 3’ side (Fig 3). Due to the unwanted effect of this recombination on phylogenetic analysis, BG35 was removed from the global alignment for the remaining analyses. These analyses suggest a possible HAstV-MAstV recombination event occurred either during or prior to infection in the NHP. Potential human-mammalian recombination events have also been reported in piglets in Colombia [10] and intriguingly in a California sea lion [11].

### In contrast to bat AstVs, NHP AstVs are distributed throughout the *Astroviridae* family

Based on the high prevalence rate and genetic diversity of AstV detected in bats compared to other species, bats have been proposed as a pimary natural reservoir for AstVs and possibly as host of the most recent conmon ancestor of the HAstVs[19,28,29]. However, with few exceptions, the bat sequences cluster within bat-specific genogroups (*MAstV* 12, 14–19) [6]. In contrast, the NHP-derived sequences are distributed throughout *Astroviridae*. Thus, to compare AstV diversity in NHP to that in bats, we performed phylogenetic analysis of sequences from these two hosts in comparison to sequences from other MAstV and AAstV hosts. These analyses took place on two distinct phylogenetic trees. Both trees were built in part from a core set of mammalian and avian AstVs reference sequences making up a reference community but differed in that one was built from the references sequences with the NHP AstV sequences, while the other was built with the bat AstV sequences.

**Fig 4:**
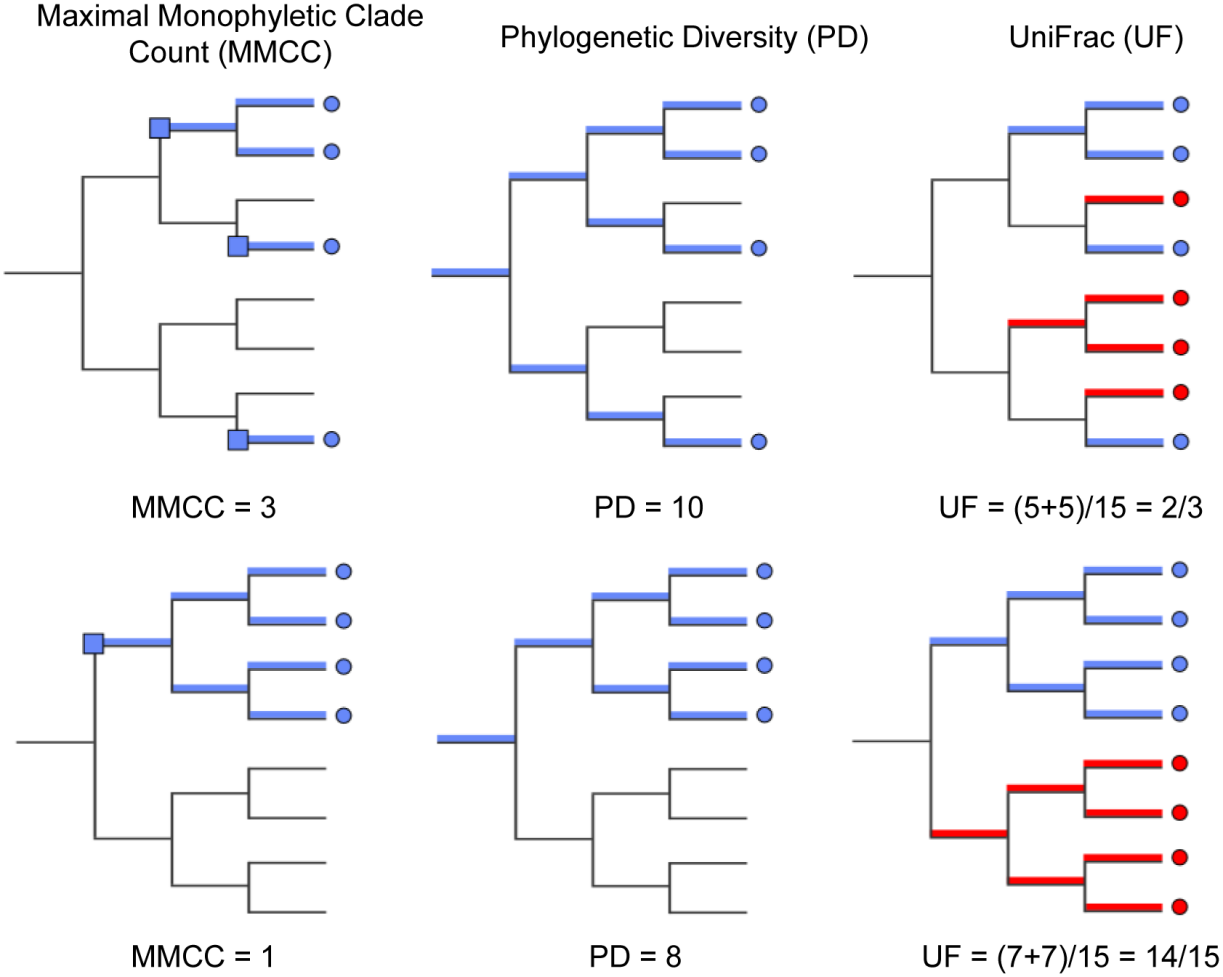
Diversity metrics illustrated graphically for a given phylogenetic tree and tip labeling. The tips of the focus community are colored blue, and represent either the monkey or bat virus population. The uncolored or red tips represent the reference community. Branches in each case are colored according to whether and how the corresponding branch lengths would be counted for the given metric. The descriptions of each metric and how they are computed are presented in Results.

On each of these trees we computed three diversity metrics. The first is the *phylogenetic diversity* metric [30], which measures the sum of branch lengths contained within the minimal subtree spanning all tips of interest as depicted in (Fig 4). Intuitively, the higher this value is, the more diverse the community. The remaining two metrics were chosen to characterize the distribution of host diversity in relation to the reference community. Of these, the first is the UniFrac [31] distance between sequences from a given host to the collection of reference sequences. This metric is computed as the sum of branch lengths unique to one community or the other, divided by the sum of all branch lengths as depicted in (Fig 4). This effectively gives us a measure of how different the viral community in question is from the reference community. Smaller or larger values indicate the community of interest is phylogenetically closer or more distant (respectively) to the reference community. Lastly, we evaluated the *maximal monophyletic clade count* of a given set of sequences on the tree as the number of unique places on the reference tree at which the sequences in question branch off (Fig 4). This measures how interspersed the sequences in question are among the reference community.

**Fig 5:**
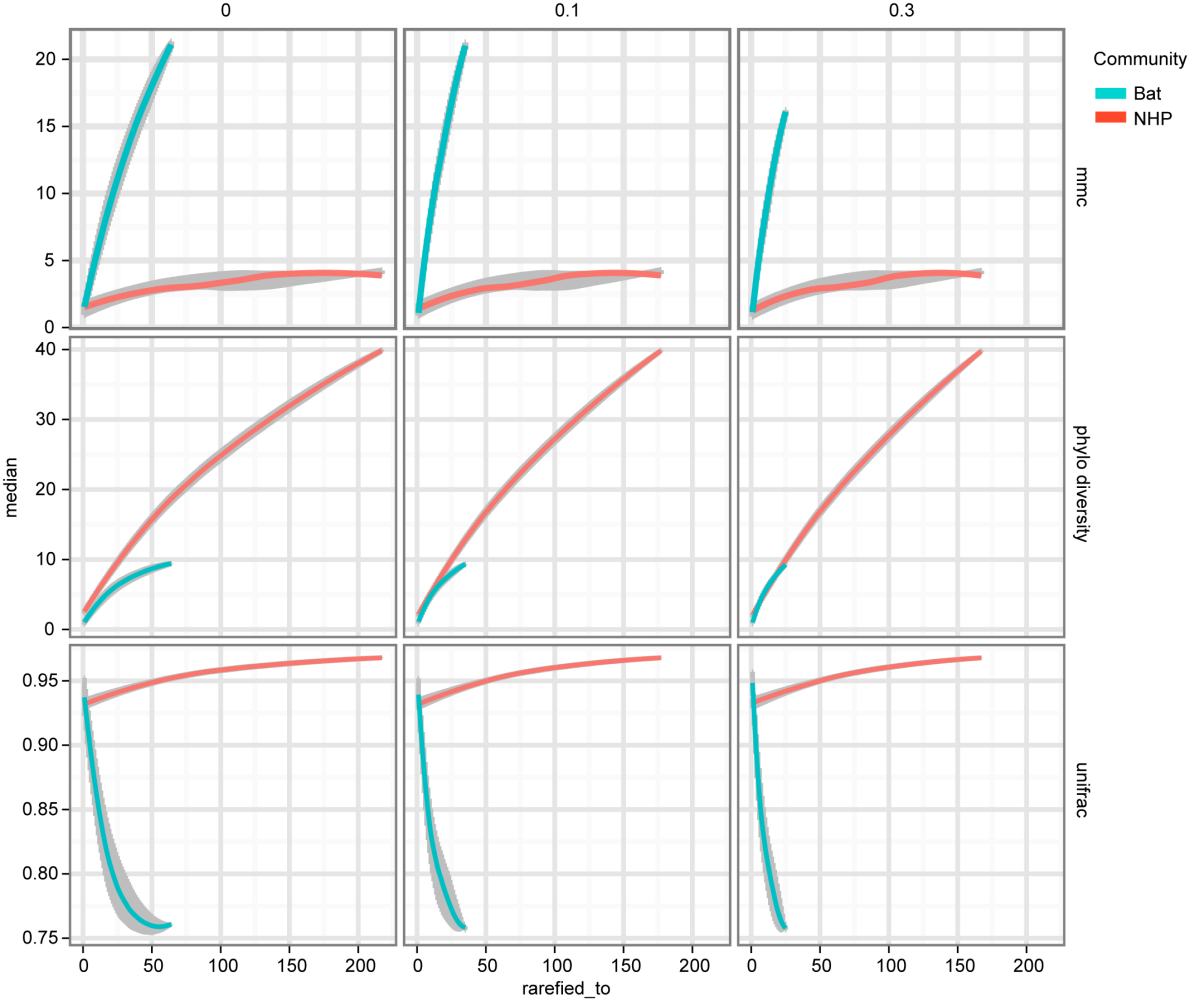
NHP and Bat AstV diversity are differently distributed with respect to the diversity of other canonical AstVs. As described in Results, MMCC, Phylogenetic Diversity (PD) and UniFrac metrics are computed for each of two phylogenetic trees—one built with NHP AstV sequences, the other bat AstV sequences, both with a shared set of “reference community” astroviruses. The columns of plots represent different clustering thresholds meant to account for novelty bias in bat sequence submissions, while the x-axis represents subsampling to account for differences in sampling depth between the two populations. As we attempt to correct for the possibility of novelty bias, the phylogenetic diversity of the NHP AstVs begins to match that of the bat AstVs. The MMCC and UniFrac metrics indicate that across subsampling depths and novelty bias correction levels, bat AstVs are on average more distant from reference community viruses, and form more isolated clades. The error bars and central points are computed via the 50% confidence interval quantiles and medians from the random subsamples, and smoothed using R’s LOESS fitting.

The first column of Fig 5 shows that, assuming bat and NHP sequences are equally unbiased representations of the corresponding viral populations, the bat AstV population has considerably higher phylogenetic diversity. However, as we begin to account for novelty bias and sampling depth by clustering sequences at various thresholds and picking representatives (as described in Materials and Methods and Discussion sections respectively), we find that NHP AstV diversity begins to match and even slightly exceed that of the bat AstVs (Fig 5). However, across all clustering and sampling depths explored, the UniFrac and MMC measurements indicate NHP AstVs are much closer to the rest of the phylogenetic tree than are the bat AstVs and more broadly distributed across the tree. In contrast, the bat AstV are less well integrated into the reference community, and more isolated from the rest of the AstV diversity. These data suggest that while bats and NHP harbor similarly diverse AstV, NHP AstV bears much greater resemblance to the community of other mammalian AstVs.

## Discussion

Our studies provide important new information that human AstVs can be detected in non-human primates. Several studies demonstrated that a single host species may be susceptible to divergent AstV genotypes including pigs, bats, California sea lions, sheep, mink, turkeys, and humans, which can be infected with strains genetically related to animal AstVs [7,8]. However, identification of mammals with AstV viruses associated with human infections has never been documented until now. Not only did we find evidence of diverse AstVs genotypes in fecal samples, we also detected AstV-specific antibodies in plasma samples. Although we did identify one NHP that was both serologically and RT-PCR-positive for HAstV-1, most of the serologically positive animals were RT-PCR negative at the time of sample collection. This suggests a previous exposure to either the same or different AstV genotype. In support of this, we identified two primates that were serologically positive for different genotypes to those detected by RT-PCR; in one case serologically MLB-positive and while HAstV-1 positive by RT-PCR and in the second serologically HAstV-1 positive while an avian AstV was detected. Future studies will have to address whether detection of AstV in non-human primates is associated with actual infection, and if so, whether such infections are asymptomatic or associated with clinical disease; none of the NHP sampled in this study appeared to have clinical disease (i.e. diarrhea) at the time of sampling.

One limitation of our study is that the genetic analysis is based on ~400 nucleotide region of the RdRp [19]. While this is the most conserved region of the AstV genome supporting the proposed genogroup assignments, precise genotyping is ideally done with full genome or at least the full ORF2 (capsid) sequence. Attempts to isolate viruses or construct full genomic sequences through a variety of methods including genome walking with degenerate primers, 3’RACE, and deep sequencing methodologies similar to those used for the novel human AstV strains [20] was unsuccessfully undertaken on all RdRp-positive samples. It is not surprising that virus isolation was unsuccessful given that very few AstVs can be cultured. However, the inability to identify further genetic sequences despite all efforts was frustrating and suggests that the amount of virus being “shed” by primates was at a very low level or that sample quality was not ideal. In spite of these limitations, we did sequence ~130 nucleotides from the conserved 5’ end of the MAstV/Hanuman langur/BG569/Bangladesh/2008 capsid that shared 77% similarity with bovine AstV (S1 Fig) and ~300 nucleotides from the conserved 5’ end of MAstV/Hoolock gibbon/Bangladesh/BG36/2007 capsid gene demonstrating that it was similar to human MLB viruses. We also obtained a larger capsid sequence from MAstV/Rhesus macaque/Bangladesh/BG31/2007. Phylogenetic analysis suggests that while unique, it is part of a subclade comprised of diverse animal hosts including cows, pigs and deer.

Bats are known to harbor a diversity of AstV species; although these viruses are unique to the bat host and haven’t been found in other species to date [19]. Nor have AstV typically associated with other animal hosts been identified in bats. In contrast, NHP harbor AstV associated with diverse animal hosts. Phylogenetic analyses comparing the sequence diversity observed in these two host groups revealed that NHP viruses exhibit a comparable level of diversity to those reported in bats. However, the distribution is quite different. In particular, AstV sequences from bats were found to be more phylogenetically isolated from AstV detected in known host species, while those of NHP are relatively well integrated within the greater phylogenetic tree. Note that these comparisons are complicated by two aspects of the sampling. The first is sampling depth. Ours is the first and only study of AstV diversity in NHP, from which 68 nucleotide sequences have been obtained. In contrast, there have been extensive studies of AstV in bats, from which hundreds of AstVs have been sequenced. To account for this, we compared randomly selected subsamples from the phylogenetic trees to see how sampling depth affected the diversity metrics, an analytic technique known as rarefaction [32]. Secondly, it is clear from the papers detailing studies in bats that there is diversity-selection bias regarding what nucleotide sequences are submitted to online repositories. For instance, in Zhu et al. AstV was detected in a total of 224 animals, while only 76 sequences were submitted to GenBank [28]. The authors did not indicate the criteria used in selecting these representatives, nor how many sequences obtained were identified with each representative submitted. As such, we have no way of precisely replicating this with our NHP AstV sequences. This problem is compounded by the fact that, in general, researchers from different studies are more likely to report sequences to GenBank if they appear to be novel. Thus, taking sequences from such a repository as a representation of the viral diversity found within these hosts will inevitably introduce a bias towards increased diversity. To account for this, we performed sequence clustering of both bat and NHP sequences at various thresholds as described in Materials and Methods, and picked one sequence per cluster for each of our comparisons. While this potentially leaves out fine grained information about abundance of AstV species or subspecies, as we increase the clustering threshold, it at least puts the communities closer to the same ground by being similarly biased towards novel selection. It is worth noting that the sequence rarefaction described above was performed after this clustering step. Cross-study comparisons would be significantly eased by thorough reporting and submission of sequence data.

Despite these caveats, the trends that emerge are clear and suggest different ecological roles for bats and NHP in the maintenance of AstV diversity. While bats appear to have sustained a distinct and robust virus population for some time, the phylogenetic distance between these viruses and those of the reference community suggest transmission to and from other organisms is relatively rare. Thus, if bats are a significant reservoir of AstV diversity that occasionally spills over into other host groups, the significance of this role must be limited to a larger timescale. In contrast, the interspersedness of the NHP AstVs within the reference community suggests the net frequency of AstV transmission to and from NHP is much higher than in bats. However, the directionality of these transmission events is unknown, and it is unclear whether NHP are capable of sustaining AstV infections without periodic reintroduction from other host groups. Thus, whatever role NHP play in the ecology of AstVs, it appears their diversity is more pertinent to the recent history and dynamism of AstVs both on a whole, and specifically as is relevant to human AstV. We are currently working on analyses directly modeling the transmission of viruses between different host groups, which we hope will shed light on some of the details of this picture which remain unclear.

The presence of recombination among the NHP RdRp sequences raises further questions about the role NHP play in the ecology of AstV. Unfortunately, it is impossible to determine what host species facilitated the recombination event responsible for the recombinant virus sampled from BG35. However, the high prevalence of human AstV among NHP, coupled with the other parental strain most closely matching sequences only observed in other NHP, particularly BG31, BG41, and MBG260 suggests that the recombination event may have occurred in NHP. Even if the recombination event did occur in another host species, the role NHP may play in the emergence of novel AstV diversity is still called into question by the unique susceptibility of NHP to such diverse AstV genotypes. This study raises important questions as to the frequency of AstV recombination within NHP hosts, and highlights the importance of continued monitoring of AstV within NHP.

In summary, a myriad of MAstV and AAstV genotypes can be detected in NHP. This further dispels the dogma that astroviruses are species-specific, and raises important questions about the role of NHP in astrovirus ecology, particularly those NHP thriving at the human-primate interface.

## Materials and Methods

### Ethics statement

The study protocol was approved by the University of Washington Institutional Animal Care and Use Committee (4233–01) and adhered to the American Society of Primatologist Principles for the Ethical Treatment of Non-Human Primates. All non-human primates included in this study were free-ranging animals sampled in their natural habitats, as such housing, feeding and environmental enrichment were not part of this study. No animals were sacrificed as part of this study. All non-human primate handing, sedation and sampling was done by trained personnel, with animal safety and comfort as the first priority.

### Sample collection

As part of our ongoing longitudinal studies of synanthropic NHP populations in Bangladesh and Cambodia [13,17,33,34], fecal material from freshly deposited stools from multiple NHP species were collected in Bangladesh (n = 844) between 2007–2008 and 2011–2012 and Cambodia (n = 68) between 2011 and 2012. Sera samples were collected from a subset of animals in 2011–2012. All of these animals were either at the animal-human interface in areas with substantial human population densities or sampled from limited numbers of zoo and wild NHP. Trapping and sampling protocols are reported in detail in [13,18]. Species, context of human contact, and global positioning system (GPS) coordinates were recorded for each sample and are described in [18].

### RNA isolation, AstV detection and sequencing

The 2007–2008 samples were collected and processed as described [13,33] and provided as a fecal homogenate to St Jude. For the 2011–2012 samples, stool (100 μL) was homogenized in 0.89% NaCl with 0.2 mm Zirconium Oxide beads (Next Advance, Averill Park, NY, USA) followed by centrifugation to form a fecal filtrate and RNA isolated from a 50μL of this filtrate on a Kingfisher Flex Magnetic Particle Processor (Thermo Fisher Scientific, Waltham, MA, USA) using the Ambion MagMAX-96 NI/ND Viral RNA Isolation kit (Life Technologies Corporation, Grand Island, NY, USA) and screened using a pan-astrovirus reverse transcription-PCR assay targeting the RdRp gene [19]. Sanger sequencing was performed by the St Jude Hartwell Center to identify the AstV genogroup. All RdRp-positive samples were then subjected to further sequencing to generate capsid sequence. Samples were tested against either strain-specific or random hexamer primers to attempt to obtain any capsid sequence using a variety of methods including genome walking with degenerate primers, 3’RACE and deep sequencing. Primers used on specific samples are described in S2 Table.

### AstV ELISA

Sera were obtained from 138 NHP in 2011–2012, 85 of which were from animals where stool was collected for RT-PCR, and tested for antibodies to specific AstVs by ELISA as described [22]. Briefly, high binding–affinity polystyrene plates (Corning Incorporated, Corning, NY) were coated with 0.05 μg/well of purified recombinant HAstV-1, MLB1, MLB2, TAstV-2 capsid protein or BSA (negative control) and incubated overnight at 4°C. Each plate contained an individual capsid protein or BSA. Plates were washed thrice with PBS in 0.05% Tween-20 (PBST) and then blocked with 4% BSA in PBST for 2 hour at room temp. Following extensive washing, limiting dilutions of the sera (neat to 10^−4^) or rabbit polyclonal antisera to the different capsid proteins were added to the plates and incubated for 1 hour at room temp. After washing, plates were incubated with 0.05 μg/mL of HRP-conjugated anti-monkey or anti-rabbit secondary antibodies (Jackson ImmunoResearch, West Grove, PA) for 1 hour then reactivity was assessed by using an HRP substrate reagent kit (R&D Systems, Minneapolis, MN). To reaction was stopped with 2N H2SO4 and absorbance read on a Multiskan Ascent microplate spectrophotometer (ThermoFisher, Waltham, MA) at 450 nm. Samples with capsid-specific absorbance greater than three times the absorbance of the sample binding to BSA were considered positive. All samples were tested in at least triplicate and experiments repeated at least twice.

### Phylogenetic analysis

#### Reference community

AstV sequences representative of various genotypes as described in Bosch et. al. [6] were used to define the *reference community* providing a backdrop for analysis monkey and bat AstV diversity as compared to AstV diversity in other hosts. Genbank numbers are shown in the Fig.

#### Bat sequences

Bat AstV sequences were obtained from a NCBI nucleotide BLAST search against the NHP BG33 sequence, together with entrez query "bat OR Chiroptera OR Megachiroptera OR Microchiroptera OR Miniopterus OR Tylonycteris OR Acerodon", in addition to sequences from [pmid: 25034867] resulting 219 AstV bat RDRP sequences.

#### Alignment

Sequences were aligned and trimmed to equivalent start and stop positions of the corresponding reference sequence plus 15 bp on either side to prevent over-trimming. Sequences which had BLAST matches of less than 0.25 identity and any trimmed sequences less than 180 bp were not included, removing two bat and 5 reference sequences from the analysis. Trimmed sequences were aligned with reference sequences using a back-translation alignment strategy as follows: a codon level alignment was made using MACSE v0.8 [35] and frameshift mutation characters, as identified by MACSE, were replaced with ambiguous "N" characters, putting the corresponding sequences in frame. The resulting sequences were translated using seqmagick v0.6.0 [36], and aligned using MAFFT v7.058b [37] resulting in amino acid alignments that were used by seqmagick backtrans-align to produce a backtranslation alignment.

#### Phylogenetic reconstruction

Phylogenetic trees were constructed using Phyml v3.0 [38]. Trees were rooted to their midpoints using the biopython library [39], placing MAstV and AAstVs on opposite sides of the root. The reference BatAstV sequence’s tip was removed from the tree for subsequent analyses, to put the NHP and bat communities on equal footing in these comparisons.

#### Phylogenetic metrics and sub-sampling

An additional phylogenetic tree was constructed as described above, for the purpose of comparing bat and NHP AstV communities with the reference AstV community. However, to avoid putting bats and NHP on different footing, the reference BatAstV was removed. A number of phylogenetic metrics were computed on this tree under tip clustering and rarefaction to characterize the bat and NHP AstV phylogenetic diversity. Tip clustering was carried out by a custom R v3.1.2 [40] script together with the ape package v3.1–4 [41]. From each tree, a distance matrix between sequences was obtained using ape's as.dist function. This matrix was subset to columns and rows corresponding to sequences in the focus community (bat or NHP AstVs), and used as the basis for an hclust clustering. For each given distance threshold, the hclust tree was cut at that threshold and one sequence representative was taken for each resulting cluster at random. The tree, together with the names of the remaining focus community sequences, were used as input to the `newick_utils`v1.6 [42] `nw_prune`command, producing a tree with all of the reference sequences, and exactly one focus community sequence per cluster. The clustering thresholds used were 0 (no clustering), 0.01 and 0.03. For each of the cluster-reduced trees, three phylogenetic metrics were computed under rarefaction. The phylogenetic diversity metric was computed using the `phylocurve_perm`R library, and that library's internal sampling procedures were used for rarefaction. UniFrac and MMCC were computed using a custom python script utilizing the Bio.Phylo library. Within this script, each focus community of sequences was rarefied by random sub-sampling of tree tips using python's random.sample function. UniFrac and MMCC metrics were then computed on the subtrees induced by this sub-sampling. UniFrac values were computed as the UniFrac distance between the rarefied focus community sequences and the unrarefied reference community sequences within the tree. MMCC values were computed as the minimum number of clades, with tips entirely composed of focus sequences, representing all focus sequences. Note this metric is dependent on the rooting of the tree, making the rooting step described above important.

### Accession numbers

Bat AstV accession numbers used include HQ613157-HQ613171, HQ613174-HQ613175, HQ613178,EU847144-EU847154,EU847156,EU847159-EU847173,EU847175-EU847195, EU847197-EU847215,EU847217-EU847220,FJ571065-FJ571068,FJ571075,FJ571077-FJ571080, FJ571082,FJ571085-FJ571086,FJ571090-FJ571091,FJ571093-FJ571108,FJ571110-FJ571111,FJ571113,FJ571115,FJ571117-FJ571119,FJ571121-FJ571131,FJ571133-FJ571135, FJ571137,FJ571139-FJ571140,JQ814856-JQ814864,JQ814866-JQ814868,JQ814870-JQ814871, HM368168-HM368172,HM368174-HM368175,KJ571377-KJ571391,KJ571393-KJ571409,KJ571411-KJ571431. NHP AstV sequences are available at GenBank under accession numbers KT852380–KT852448.

## Supporting Information

S1 Fig BG569 capsid shares similarity to bovine AstV. Clustal W alignments on ~130 nucleotides from the ORF2 capsid gene of NHP BG569 were performed using BioEdit and MEGA6. Phylogenetic trees were constructed and evolutionary history inferred using the Neighbor-Joining method. GenBank accession numbers for the reference strains are given.
(TIF)

S1 Table Information on AstV-positive samples. (DOCX)

S2 Table Primer Information. (DOCX)

## References

[1] Matsui SM, Greenberg HB (1996) Astroviruses In: Fields BN, David PMH, Knipe M, editors. Field's Virology. 3 ed Philadelphia: Lippincott Williams and Wilkins pp. 811–824.

[2] Mendez E, Arias CF (2007) Astroviruses In: Knipe DM, Howley PM, editors. Field's Virology. 5 ed Philadelphia: Lippincott Williams and Wilkins pp. 981–1000.

[3] Guix S, Bosch A, Pinto RM (2013) Astrovirus Taxonomy In: Schultz-Cherry S, editor. Astrovirus Research. New York: Springer Science + Business Media pp. 97–118.

[4] De Benedictis P, Schultz-Cherry S, Burnham A, Cattoli G (2011) Astrovirus infections in humans and animals—molecular biology, genetic diversity, and interspecies transmissions. Infect Genet Evol 11: 1529–1544. 10.1016/j.meegid.2011.07.024 21843659 PMC7185765

[5] Mendenhall IH, Smith GJ, Vijaykrishna D (2015) Ecological Drivers of Virus Evolution: Astrovirus as a Case Study. J Virol 89: 6978–6981. 10.1128/JVI.02971-14 25948751 PMC4473558

[6] Bosch A, Pintó RM, Guix S (2014) Human Astroviruses. Clinical Microbiology Reviews 27: 1048–1074. 10.1128/CMR.00013-14 25278582 PMC4187635

[7] Jiang H, Holtz LR, Bauer I, Franz CJ, Zhao G, et al (2013) Comparison of novel MLB-clade, VA-clade and classic human astroviruses highlights constrained evolution of the classic human astrovirus nonstructural genes. Virology 436: 8–14. 10.1016/j.virol.2012.09.040 23084422 PMC3545029

[8] Cattoli G, Chu DKW, Peiris M (2013) Astrovirus infections in animal mammalian species In: Schultz-Cherry S, editor. Astrovirus Research. New York: Springer Science+Business Media pp. 135–149.

[9] Pankovics P, Boros A, Kiss T, Delwart E, Reuter G (2015) Detection of a mammalian-like astrovirus in bird, European roller (Coracias garrulus). Infect Genet Evol 34: 114–121. 10.1016/j.meegid.2015.06.020 26096774

[10] Finkbeiner SR, Le BM, Holtz LR, Storch GA, Wang D (2009) Detection of newly described astrovirus MLB1 in stool samples from children. Emerg Infect Dis 15: 441–444. 10.3201/eid1503.081213 19239759 PMC2666294

[11] Rivera R, Nollens HH, Venn-Watson S, Gulland FM, Wellehan JF Jr. (2010) Characterization of phylogenetically diverse astroviruses of marine mammals. J Gen Virol 91: 166–173. 10.1099/vir.0.015222-0 19759240

[12] Farkas T, Sestak K, Wei C, Jiang X (2008) Characterization of a Rhesus Monkey Calicivirus Representing a New Genus of Caliciviridae. Journal of Virology 82: 5408–5416. 10.1128/JVI.00070-08 18385231 PMC2395209

[13] Oberste MS, Feeroz MM, Maher K, Nix WA, Engel GA, et al (2013) Characterizing the picornavirus landscape among synanthropic nonhuman primates in Bangladesh, 2007 to 2008. J Virol 87: 558–571. 10.1128/JVI.00837-12 23097448 PMC3536389

[14] Farkas T, Cross RW, Hargitt E, Lerche NW, Morrow AL, et al (2010) Genetic Diversity and Histo-Blood Group Antigen Interactions of Rhesus Enteric Caliciviruses. Journal of Virology 84: 8617–8625. 10.1128/JVI.00630-10 20554772 PMC2919043

[15] Kalter SS (1982) Enteric viruses of nonhuman primates. Vet Pathol Suppl 7: 33–43. 6153011

[16] Jiang B, McClure HM, Fankhauser RL, Monroe SS, Glass RI (2004) Prevalence of rotavirus and norovirus antibodies in non-human primates. Journal of Medical Primatology 33: 30–33. 15061730 10.1111/j.1600-0684.2003.00051.x

[17] Feeroz MM, Soliven K, Small CT, Engel GA, Andreina Pacheco M, et al (2013) Population dynamics of rhesus macaques and associated foamy virus in Bangladesh. Emerg Microbes Infect 2: e29 10.1038/emi.2013.23 26038465 PMC3675400

[18] Karlsson EA, Engel GA, Feeroz MM, San S, Rompis A, et al (2012) Influenza virus infection in nonhuman primates. Emerg Infect Dis 18: 1672–1675. 10.3201/eid1810.120214 23017256 PMC3471624

[19] Chu DKW, Poon LLM, Guan Y, Peris JSM (2008) Novel astroviruses in insectivorous bats. J Virol 82: 9107–9114. 10.1128/JVI.00857-08 18550669 PMC2546893

[20] Finkbeiner SR, Kirkwood CD, Wang D (2008) Complete genome sequence of a highly divergent astrovirus isolated from a child with acute diarrhea. Virology Journal 5. 10.1186/1743-422X-5-117 PMC2576171 18854035

[21] Finkbeiner SR, Li Y, Ruone S, Conrardy C, Gregoricus N, et al (2009) Identification of a novel astrovirus (astrovirus VA1) associated with an outbreak of acute gastroenteritis. J Virol 83: 10836–10839. 10.1128/JVI.00998-09 19706703 PMC2753140

[22] Meliopoulos VA, Kayali G, Burnham A, Oshansky CM, Thomas PG, et al (2014) Detection of Antibodies against Turkey Astrovirus in Humans. PLoS ONE 9: e96934 10.1371/journal.pone.0096934 24826893 PMC4020816

[23] Holtz LR, Bauer IK, Jiang H, Belshe R, Freiden P, et al (2014) Seroepidemiology of Astrovirus MLB1. Clinical and Vaccine Immunology 21: 908–911. 10.1128/CVI.00100-14 24789796 PMC4054231

[24] Shan T, Li L, Simmonds P, Wang C, Moeser A, et al (2011) The fecal virome of pigs on a high-density farm. J Virol 85: 11697–11708. 10.1128/JVI.05217-11 21900163 PMC3209269

[25] Sun N, Yang Y, Wang G-S, Shao X-Q, Zhang S-Q, et al (2014) Detection and Characterization of Avastrovirus Associated with Diarrhea Isolated from Minks in China. Food and Environmental Virology 6: 169–174. 10.1007/s12560-014-9155-3 24915926

[26] Capella-Gutiérrez S, Silla-Martínez JM, Gabaldón T (2009) trimAl: a tool for automated alignment trimming in large-scale phylogenetic analyses. Bioinformatics 25: 1972–1973. 10.1093/bioinformatics/btp348 19505945 PMC2712344

[27] Bruen TC, Philippe H, Bryant D (2006) A simple and robust statistical test for detecting the presence of recombination. Genetics 172: 2665–2681. 16489234 10.1534/genetics.105.048975 PMC1456386

[28] Zhu HC, Chu DKW, Liu W, Dong BQ, Zhang SY, et al (2009) Detection of diverse astroviruses from bats in China. Journal of General Virology 90: 883–887. 10.1099/vir.0.007732-0 19264622

[29] Xiao J, Li J, Hu G, Chen Z, Wu Y, et al (2011) Isolation and phylogenetic characterization of bat astroviruses in southern China. Archives of Virology 156: 1415–1423. 10.1007/s00705-011-1011-2 21573690

[30] Faith DP (1992) Conservation evaluation and phylogenetic diversity. Biological Conservation 61: 1–10.

[31] Lozupone C, Knight R (2005) UniFrac: a New Phylogenetic Method for Comparing Microbial Communities. Applied and Environmental Microbiology 71: 8228–8235. 16332807 10.1128/AEM.71.12.8228-8235.2005 PMC1317376

[32] Simberloff D (1972) Properties of the rarefaction of diversity measurement. The American Naturalist 106: 414–418.

[33] Oberste MS, Feeroz MM, Maher K, Nix WA, Engel GA, et al (2013) Naturally acquired picornavirus infections in primates at the Dhaka zoo. J Virol 87: 572–580. 10.1128/JVI.00838-12 23097447 PMC3536375

[34] Hasan MK, Feeroz MM, Jones-Engel L, Engel GA, Kanthaswamy S, et al (2014) Diversity and molecular phylogeny of mitochondrial DNA of rhesus macaques (Macaca mulatta) in Bangladesh. Am J Primatol 76: 1094–1104. 10.1002/ajp.22296 24810278 PMC4487988

[35] Ranwez V, Harispe S, Delsuc F, Douzery EJP (2011) MACSE: Multiple Alignment of Coding SEquences Accounting for Frameshifts and Stop Codons. PLoS ONE 6: e22594 10.1371/journal.pone.0022594 21949676 PMC3174933

[36] Matsen IV FA (2012) seqmagick.

[37] Katoh K, Misawa K, Kuma Ki, Miyata T (2002) MAFFT: a novel method for rapid multiple sequence alignment based on fast Fourier transform. Nucleic Acids Research 30: 3059–3066. 12136088 10.1093/nar/gkf436 PMC135756

[38] Guindon S, Dufayard J-F, Lefort V, Anisimova M, Hordijk W, et al (2010) New Algorithms and Methods to Estimate Maximum-Likelihood Phylogenies: Assessing the Performance of PhyML 3.0. Systematic Biology 59: 307–321. 10.1093/sysbio/syq010 20525638

[39] Cock PJA, Antao T, Chang JT, Chapman BA, Cox CJ, et al (2009) Biopython: freely available Python tools for computational molecular biology and bioinformatics. Bioinformatics 25: 1422–1423. 10.1093/bioinformatics/btp163 19304878 PMC2682512

[40] R Core Team (2014) A language and environment for statistical computing. Vienna, Austria: R Foundation for Statistical Computing.

[41] Paradis E, Claude J, Strimmer K (2004) APE: Analyses of Phylogenetics and Evolution in R language. Bioinformatics 20: 289–290. 14734327 10.1093/bioinformatics/btg412

[42] Junier T, Zdobnov EM (2010) The Newick utilities: high-throughput phylogenetic tree processing in the Unix shell. Bioinformatics 26: 1669–1670. 10.1093/bioinformatics/btq243 20472542 PMC2887050

