# Supplementary figures and images for: Non-Human Primates Harbor Diverse Mammalian and Avian Astroviruses Including Those Associated with Human Infections
Source: PLoS Pathog. 2015 Nov 16;11(11):e1005225. doi: 10.1371/journal.ppat.1005225 (PMC4646697; doi:10.1371/journal.ppat.1005225)

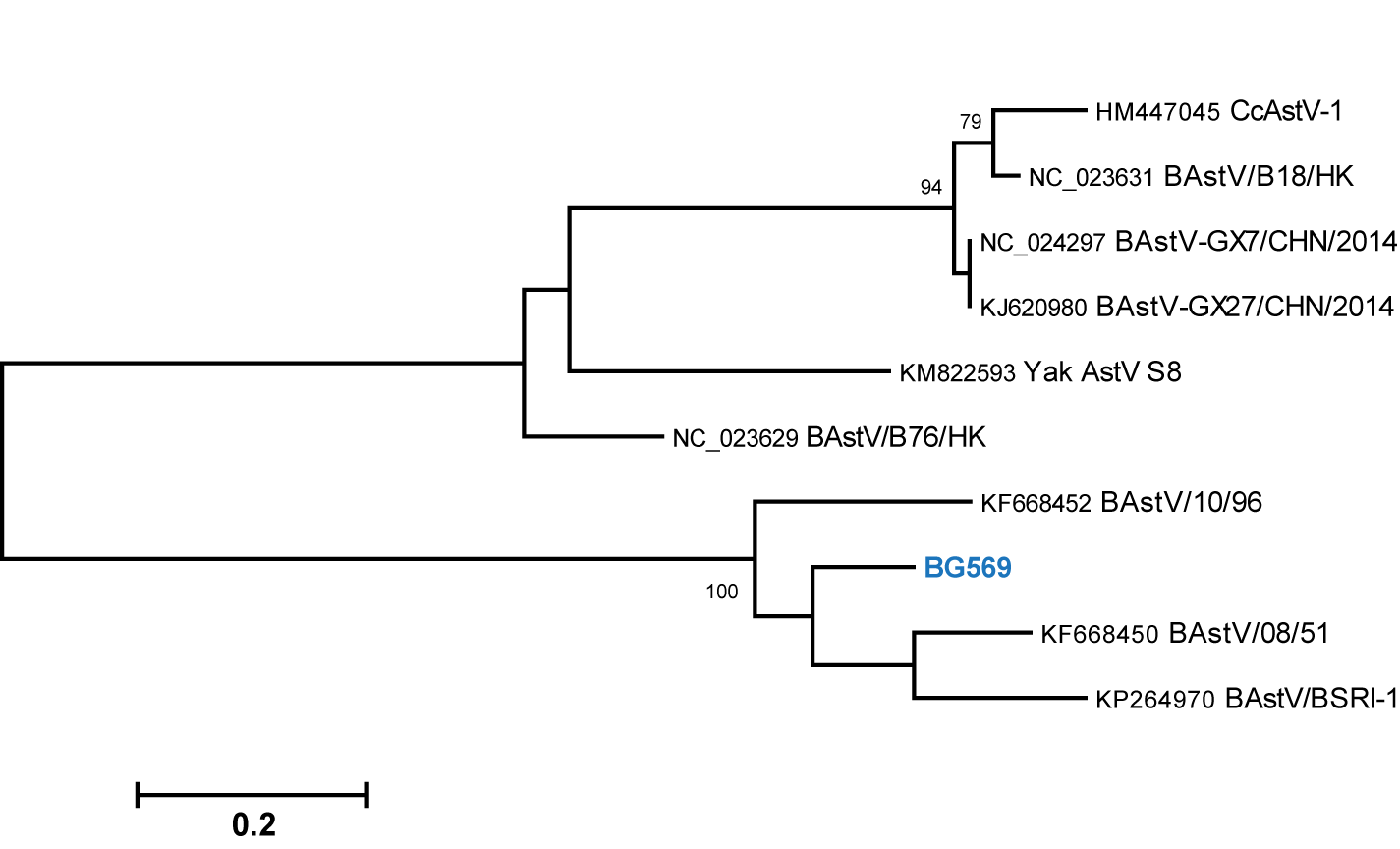

Supplement: S1 Fig — Clustal W alignments on ~130 nucleotides from the ORF2 capsid gene of NHP BG569 were performed using BioEdit and MEGA6. Phylogenetic trees were constructed and evolutionary history inferred using the Neighbor-Joining method. GenBank accession numbers for the reference strains are given. (TIF) [file ppat.1005225.s001.tif]
