# Supplementary material for: Non-Human Primates Harbor Diverse Mammalian and Avian Astroviruses Including Those Associated with Human Infections
Source: PLoS Pathog. 2015 Nov 16;11(11):e1005225. doi: 10.1371/journal.ppat.1005225 (PMC4646697; doi:10.1371/journal.ppat.1005225)
Supplement: S1 Table — (DOCX) [file ppat.1005225.s002.docx]

| Supplementary Table 1. Information on AstV-positive samples | | | | | | |
| --- | --- | --- | --- | --- | --- | --- |
| Sample ID | NHP | Country | Context | RdRp Genotype (% similarity) | Proposed AstV Nomenclature | ELISA Result |
| BG4 | Rhesus | Bangladesh | Urban | HAstV1  (87%) | MAstV/Rhesus macaque/Bangladesh/  BG4/2007 | NSA^1^ |
| BG12 | Rhesus | Bangladesh | Urban | HAstV1  (88%) | MAstV/Rhesus macaque/Bangladesh/  BG12/2007 | NSA |
| BG21 | Rhesus | Bangladesh | Performing | HAstV1  (88%) | MAstV/Rhesus macaque/Bangladesh/  BG21/2007 | NSA |
| BG23 | Rhesus | Bangladesh | Urban | HAstV1  (88%) | MAstV/Rhesus macaque/Bangladesh/  BG23/2007 | NSA |
| BG26 | Rhesus | Bangladesh | Temple | HAstV1  (87%) | MAstV/Rhesus macaque/Bangladesh/  BG26/2007 | NSA |
| BG28 | Rhesus | Bangladesh | Temple | HAstV1  (88%) | MAstV/Rhesus macaque/Bangladesh/  BG28/2007 | NSA |
| BG31 | Rhesus | Bangladesh | Temple | Unique  (n/a^2^) | MAstV/Rhesus macaque/Bangladesh/  BG31/2007 | NSA |
| BG33 | Rhesus | Bangladesh | Temple | Unique  (n/a) | MAstV/Rhesus macaque/Bangladesh/  BG33/2007 | NSA |
| BG35 | Rhesus | Bangladesh | Temple | HAstV1  (62%) | MAstV/Rhesus macaque/Bangladesh/  BG35/2007 | NSA |
| BG36 | Hoolock gibbon | Bangladesh | Wild | MLB1  (83%) | MAstV/Hoolock gibbon/Bangladesh/  BG36/2007 | NSA |
| BG41 | Capped langur | Bangladesh | Wild | Unique  (n/a) | MAstV/Capped langur/Bangladesh/  BG41/2007 | NSA |
| BG44 | Capped langur | Bangladesh | Wild | MLB1  (84%) | MAstV/Capped langur/Bangladesh/  BG44/2007 | NSA |
| BG59 | Rhesus | Bangladesh | Urban | HAstV1  (87%) | MAstV/Rhesus macaque/Bangladesh/  BG59/2007 | NSA |
| BG63 | Rhesus | Bangladesh | Urban | HAstV1  (87%) | MAstV/Rhesus macaque/Bangladesh/  BG63/2007 | NSA |
| BG71 | Rhesus | Bangladesh | Urban | HAstV1  (87%) | MAstV/Rhesus macaque/Bangladesh/  BG71/2007 | NSA |
| BG74 | Rhesus | Bangladesh | Urban | HAstV1  (86%) | MAstV/Rhesus macaque/Bangladesh/  BG74/2007 | NSA |
| BG87 | Rhesus | Bangladesh | Urban | HAstV1  (75%) | MAstV/Rhesus macaque/Bangladesh/  BG87/2007 | NSA |
| BG108 | Rhesus | Bangladesh | Wild | HAstV1  (86%) | MAstV/Rhesus macaque/Bangladesh/  BG108/2007 | NSA |
| BG113 | Rhesus | Bangladesh | Urban | Unique  (n/a) | MAstV/Rhesus macaque/Bangladesh/  BG113/2007 | NSA |
| BG114 | Rhesus | Bangladesh | Urban | HAstV1  (83%) | MAstV/Rhesus macaque/Bangladesh/  BG114/2007 | NSA |
| BG124 | Rhesus | Bangladesh | Urban | HAstV1  (87%) | MAstV/Rhesus macaque/Bangladesh/  BG124/2007 | NSA |
| BG132 | Rhesus | Bangladesh | Urban | HAstV1  (78%) | MAstV/Rhesus macaque/Bangladesh/  BG132/2007 | NSA |
| MBG23 | Rhesus | Bangladesh | Urban | HAstV1  (87%) | MAstV/Rhesus macaque/Bangladesh/  MBG23/2007 | NSA |
| MBG31 | Rhesus | Bangladesh | Urban | VA2  (82%) | MAstV/Rhesus macaque/Bangladesh/  MBG31/2007 | NSA |
| BG302 | Rhesus | Bangladesh | Temple | HAstV1  (87%) | MAstV/Rhesus macaque/Bangladesh/  BG302/2007 | NSA |
| BG309 | Rhesus | Bangladesh | Temple | HAstV1  (87%) | MAstV/Rhesus macaque/Bangladesh/  BG309/2007 | NSA |
| BG313 | Rhesus | Bangladesh | Temple | HAstV1  (87%) | MAstV/Rhesus macaque/Bangladesh/  BG313/2007 | NSA |
| BG322 | Rhesus | Bangladesh | Temple | HAstV1  (87%) | MAstV/Rhesus macaque/Bangladesh/  BG322/2007 | NSA |
| BG324 | Rhesus | Bangladesh | Temple | HAstV1  (88%) | MAstV/Rhesus macaque/Bangladesh/  BG324/2007 | NSA |
| BG329 | Rhesus | Bangladesh | Temple | HAstV1  (87%) | MAstV/Rhesus macaque/Bangladesh/  BG329/2007 | NSA |
| BG330 | Rhesus | Bangladesh | Temple | HAstV1  (87%) | MAstV/Rhesus macaque/Bangladesh/  BG330/2007 | NSA |
| BG353 | Rhesus | Bangladesh | Urban | HAstV1  (87%) | MAstV/Rhesus macaque/Bangladesh/  BG353/2008 | NSA |
| BG360 | Rhesus | Bangladesh | Urban | Canine  (77%) | MAstV/Rhesus macaque/Bangladesh/  BG360/2008 | NSA |
| MBG34 | Rhesus | Bangladesh | Performing | HAstV1  (87%) | MAstV/Rhesus macaque/Bangladesh/  MBG34/2008 | NSA |
| BG391 | Rhesus | Bangladesh | Temple | HAstV1  (87%) | MAstV/Rhesus macaque/Bangladesh/  BG391/2008 | NSA |
| BG410 | Rhesus | Bangladesh | Tea garden | Unique  (n/a) | MAstV/Rhesus macaque/Bangladesh/  BG410/2008 | NSA |
| BG411 | Rhesus | Bangladesh | Tea garden | HAstV1  (87%) | MAstV/Rhesus macaque/Bangladesh/  BG411/2008 | NSA |
| BG418 | Capped langur | Bangladesh | Tea garden | HAstV1  (87%) | MAstV/Capped langur/Bangladesh/  BG418/2008 | NSA |
| BG463 | Rhesus | Bangladesh | Urban | PAstV  (65%) | MAstV/Rhesus macaque/Bangladesh/  BG463/2008 | NSA |
| BG469 | Rhesus | Bangladesh | Urban | PAstV  (65%) | MAstV/Rhesus macaque/Bangladesh/  BG469/2008 | NSA |
| BG481 | Rhesus | Bangladesh | Urban | Canine  (80%) | MAstV/Rhesus macaque/Bangladesh/  BG481/2008 | NSA |
| BG482 | Rhesus | Bangladesh | Urban | PAstV  (62%) | MAstV/Rhesus macaque/Bangladesh/  BG482/2008 | NSA |
| BG502 | Hamadryus baboon | Bangladesh | Zoo | AAstV  (53%) | AAstV/Hamadryus baboon/Bangladesh/  BG502/2008 | NSA |
| BG506 | Vervet | Bangladesh | Zoo | MLB1  (79%) | MAstV/Vervet/Bangladesh/BG506/2008 | NSA |
| BG507 | Olive baboon | Bangladesh | Zoo | MLB1  (79%) | MAstV/Olive baboon/Bangladesh/  BG507/2008 | NSA |
| BG531 | Rhesus | Bangladesh | Urban | Canine  (84%) | MAstV/Rhesus macaque/Bangladesh/  BG531/2008 | NSA |
| BG552 | Hanuman langur | Bangladesh | Urban | HAstV1  (86%) | MAstV/Hanuman langur/Bangladesh/  BG552/2008 | NSA |
| BG553 | Hanuman langur | Bangladesh | Urban | HAstV1  (79%) | MAstV/Hanuman langur/Bangladesh/  BG553/2008 | NSA |
| BG556 | Hanuman langur | Bangladesh | Urban | MLB2 | MAstV/Hanuman langur/Bangladesh/  BG556/2008 | NSA |
| BG569 | Hanuman langur | Bangladesh | Urban | Unique  (n/a) | MAstV/Hanuman langur/Bangladesh/  BG569/2008 | NSA |
| BG573 | Rhesus | Bangladesh | Urban | HAstV1  (87%) | MAstV/Rhesus macaque/Bangladesh/  BG573/2008 | NSA |
| BG574 | Rhesus | Bangladesh | Urban | Ovine  (69%) | MAstV/Rhesus macaque/Bangladesh/  BG574/2008 | NSA |
| BG581 | Rhesus | Bangladesh | Urban | Ovine  (69%) | MAstV/Rhesus macaque/Bangladesh/  BG581/2008 | NSA |
| BG642 | Rhesus | Bangladesh | Zoo | AAstV  (53%) | AAstV/Rhesus macaque/Bangladesh/  BG642/2008 | NSA |
| BG717 | Rhesus | Bangladesh | Temple | HAstV1  (87%) | MAstV/Rhesus macaque/Bangladesh/  BG717/2008 | NSA |
| BG735 | Rhesus | Bangladesh | Temple | HAstV1  (85%) | MAstV/Rhesus macaque/Bangladesh/  BG735/2008 | NSA |
| MBG 37-42(3) | Hanuman langur | Bangladesh | Urban | HAstV1  (87%) | MAstV/Rhesus macaque/Bangladesh/  MBG37-42(3)/2008 | NSA |
| MBG 46-49(3) | Hanuman langur | Bangladesh | Urban | HAstV1  (87%) | MAstV/Rhesus macaque/Bangladesh/  MBG46-49(3)/2008 | NSA |
| WR10 | Gibbon | Cambodia |  | HAstV1  (87%) | MAstV/Gibbon/  Cambodia/WR10/2011 | NSA |
| MBG218 | Rhesus | Bangladesh | Performing | HAstV  (87%) | MAstV/Rhesus macaque/Bangladesh/  MBG218/2012 | Positive (HAstV1) |
| MBG230 | Rhesus | Bangladesh | Urban | HAstV1  (87%) | MAstV/Rhesus macaque/Bangladesh/  MBG230/2012 | Negative |
| MBG248 | Rhesus | Bangladesh | Temple | HAstV1  (87%) | MAstV/Rhesus macaque/Bangladesh/  MBG248/2012 | Positive (MLB) |
| MBG260 | Rhesus | Bangladesh | Temple | Unique  (n/a) | MAstV/Rhesus macaque/Bangladesh/  MBG260/2012 | Negative |
| MBG267 | Rhesus | Bangladesh | Temple | HAstV1  (58%) | MAstV/Rhesus macaque/Bangladesh/  MBG267/2012 | Negative |
| FCB5 | Long-tailed | Cambodia | Urban | Unique  (n./a) | MAstV/Long-tailed macaque/Cambodia/  FCB5/2011 | NSA |
| FCB17 | Long-tailed | Cambodia | Temple | AAstV  (59%) | AAstV/Long-tailed macaque/Cambodia/  FCB17/2011 | NSA |
| MCB35 | Long-tailed | Cambodia | Urban | Unique  (n/a) | MAstV/Long-tailed macaque/Cambodia/  MCB35/2011 | Positive (HAstV1) |
| MCB 37 | Long-tailed | Cambodia | Urban | Unique  (n/a) | MAstV/Long-tailed macaque/Cambodia/  MCB37/2011 | Negative |
| MCB 43 | Long-tailed | Cambodia | Temple | AAstV  (54%) | AAstV/Long-tailed macaque/Cambodia/  MCB43/2011 | Positive (HAstV1) |

^1^No sera available; ^2^Not available
