# Supplementary material for: Non-Human Primates Harbor Diverse Mammalian and Avian Astroviruses Including Those Associated with Human Infections
Source: PLoS Pathog. 2015 Nov 16;11(11):e1005225. doi: 10.1371/journal.ppat.1005225 (PMC4646697; doi:10.1371/journal.ppat.1005225)
Supplement: S2 Table — (DOCX) [file ppat.1005225.s003.docx]

| **Samples** | **Primer number** | **Primer Location** | **Sequence 5'-3'** |
| --- | --- | --- | --- |
| ALL samples | 1 | RdRP | GARTTYGATTGGRCKCGKTAYGA |
| ALL samples | 2 | RdRP | GARTTYGATTGGRCKAGGTAYGA |
| ALL samples | 3 | RdRP | GGYTTKACCCACATNCCRAA |
| ALL positive samples | 4 | RdRP | TTYGGNATGTGGGTMAARCC |
| ALL samples | 5 | RdRP | CGKTAYGATGGKACKATHCC |
| ALL samples | 6 | RdRP | AGGTAYGATGGKACKATHCC |
| MBG230 | 7 | RdRP | CTTTGGAGGGGHGGACCAA |
| BG31 | 8 | Capsid | TGGTCAACTATTGGCTCCAAGCCT |
| BG31 | 9 | Capsid | ACCAGACAACTACGTTTCCCTCGT |
| BG31 | 10 | Capsid | ACACTACGAAAGTGGTGGTCCAGT |
| BG31 | 11 | Capsid | TCAATATAATCCTTGAAAGGATGAT |
| BG569 | 12 | Capsid | GGWCCWGGRGTRAKCTGCTG |
| BG324, BG35, MBG230, MBG260 | 13 | Capsid | CCTGTAAAGCACCACTCTG |
| BG124 | 14 | Capsid | CGNCGGCTNTCNCAGATNGTT |
| BG31 | 15 | Capsid | GAGTGACCGCGGCCGCTTTTTTTTTTTTTTTTTTTT |
| MGB31b | 16 | Capsid | TCCAATTTTTCCAATTGTTGC |
| BG36 | 17 | Capsid | TATATGGTTTTAGTTGGAGTTCAC |
| BG36 | 18 | Capsid | GCATACATTTATGCTGGAAGAAAA |
| MBG218 | 19 | Random primer | d(TTTTTTTTTTTTTTTTTT) |

**Supplemental Table 2**: Primer Information

| **Primer references:** |  |  |  |
| --- | --- | --- | --- |
| 1,2, 3, 5, 6 | Chu, D. K. W., Poon, L. L. M., Guan, Y. & Peiris, J. S. M. (2008). Novel astroviruses in insectivorous bats. J Virol 82, 9107–9114 | | |
|  |  |  |  |
| 4,7,8,9,10,11,12,13,14,16,17,18 | Lab designed primers | | |
| 15 | Wang QH, Han MG, Cheetham S, Souza M, Funk JA, Saif LJ. 2005. Porcine noroviruses related to human noroviruses. Emerg. Infect. Dis. 11:1874 –1881 | | |
| 19 | Life Technologies, Grand Island, NY | | |
